# Process evaluation of integrated diabetes management at primary healthcare facilities in Pakistan: a mixed-methods study

**DOI:** 10.3399/bjgpopen18X101612

**Published:** 2018-11-14

**Authors:** Muhammad Amir Khan, John D Walley, Saima Ali, Rebecca King, Shaheer Ellahi Khan, Nida Khan, Faisal Imtiaz Sheikh, Haroon Jehangir Khan

**Affiliations:** 1 Chief Coordinating Professional, Association for Social Development, Islamabad, Pakistan; 2 Professor of International Public Health, Nuffield Centre for International Health and Development, Leeds Institute of Health Sciences, University of Leeds, Leeds, UK; 3 Research Coordinator, Association for Social Development, Islamabad, Pakistan; 4 Lecturer, Nuffield Centre for International Health and Development, Leeds Institute of Health Sciences, University of Leeds, Leeds, UK; 5 Assistant Professor, Humanities and Social Sciences Department, Bahria University, Islamabad, Pakistan; 6 Project Coordinator, Association for Social Development, Islamabad, Pakistan; 7 Research Coordinator, Association for Social Development, Islamabad, Pakistan; 8 Director, NCD & Mental Health, Directorate General of Health Services, Punjab, Pakistan

## Abstract

**Background:**

Integrated care for diabetes and associated conditions at primary level health facilities can make care available to a much larger population, especially in rural areas.

**Aim:**

This process evaluation was to understand how the authors' integrated care was implemented and experienced by the care providers and patients, and to inform modifications prior to province-wide scale-up.

**Design & setting:**

The mixed-method study was conducted as part of a cluster randomised trial on integrated diabetes care at 14 public health facilities.

**Method:**

The care practices were assessed by analysing the routine clinical records of 495 registered patients with diabetes. Then semi-structured interviews with service providers and patients were used to understand their respective care experiences. A framework approach was applied to analyse and interpret the qualitative data.

**Results:**

The intervention and the study were implemented as intended under routine conditions in rural health centres. Key service processes effectively delivered included: skill-based training; screening and diagnostic tests; treatment card records; and the additional case management as per desk guide, including monitoring progress in glucose and weight at follow-up consultations, and mobile phone calls to help adherence. However, social and cultural factors affected clients' ability to change lifestyles, especially for women. The intervention effect was limited by the short study follow-up of only 9 months.

**Conclusion:**

Integrated diabetes care was feasible, both for providers and patients, and potentially scalable at primary care facilities under routine conditions in Pakistan. Additional operational interventions are required for sustained drug supplies, supervision, in-service training, and to address the social challenges to healthy activity and eating, especially for women.

## How this fits in

The delivery of integrated diabetes care at public facilities has never been evaluated in Pakistan. The process evaluation of early implementation was to help understanding and refining of the delivery of care tasks in routine healthcare settings.

## Introduction

To achieve better coverage, early diagnosis, and continuity of care, the World Health Organization recommends^1^ integrated delivery of diabetes care at primary and secondary level health care; implying delivering diabetes care as part of routine outpatient and community-based care. The challenge is how to achieve this in resource-poor settings. Pakistan has the seventh highest burden of diabetes mellitus in the world.^2^ The burden of disease is expected to increase if adequate control measures are not taken.^3^ High diabetes prevalence poses a challenge to economic development^4^ and to the quality of life of the population.^5^

In Pakistan, integrated diabetes care is to be delivered by medical and allied staff already working at health centres and sub-district hospitals. These publicly funded health facilities provide free-of-charge basic clinical and diagnostic services to defined catchment populations. However, lack of contextualised programme guidelines and tools to diagnose, treat, and educate patients with diabetes results in widely varied and poor quality care provision and an inadequate patient response to the care.^6^

To address the varied quality of care, the Provincial Non-Communicable Disease (NCD) Programme and partners developed three products by adapting international best practice guidelines.^7–11^ These are: a case management desk-guide; a lifestyle modification counselling tool; and a training package for care providers. The products were aimed at enhancing the diabetes care practices and achieving better glycaemic control. In light of other reported experiences,^12^ this study's enhanced care intervention covered the screening, diagnosis, prescription, education, and follow-up of patients with diabetes.

The trial design and findings are to be published in this journal (in press).^13^ The key trial finding was that the enhanced care intervention improved the glucose control results. The enhanced care patients achieved a higher proportion of desirable blood glucose level of HbA1c ≤7%, and a higher mean patient difference between their baseline and end-line HbA1c. However, after adjusting for between-cluster variation, the intervention effect has a 17% probability of being by chance (*P* = 0.17).

Process evaluation of complex intervention is important to understand how context, issues of fidelity, and participant interactions with, and responses to, the intervention affect outcomes.^14^ This process evaluation uses quantitative and qualitative methods in a sequence^15^ to: assess fidelity to the planned intervention by exploring how it was implemented; explore the ways in which providers and patients experienced the intervention; and inform modifications to the intervention before scaling.

## Method

Routine clinical care data (quantitative) were used to assess the fidelity to the care protocols (intervention). The qualitative interviews were designed to explore the ways in which providers and patients responded to, and interacted with, the intervention.^16^ The data collection and analysis were organised around five key intervention components:

- patient identification and diagnosis;
- treatment and record-keeping;
- lifestyle modification;
- patient follow-up and referral; and
- material inputs for patient care (see Box 1).

A pragmatic approach to the research^17^ was adopted, through which the authors aimed to make informed policy and practice recommendations for the NCD Programme in Punjab province. Box 2 shows the logic model for the intervention.

**Box 1.** Selected care tasks and key indicators

Care task	Key indicators	
	Quantitative	Qualitative
Screening and diagnosis	1. Number of patients with diabetes registered, as percentage of overall outpatient attendance2. Number and percentage of patients examined for baseline clinical and laboratory, and age/anthropometric measurements	Patient’s and provider’s experiences, as well as practice deviations and reasons for: identifying and examining patients who are overweight or symptomatic of diabetes mellitus conducting clinical and laboratory exams, and diagnosis
Prescriptions	3. Number and percentage of patients prescribed as per guidelines for diabetes and/or hypertension: without comorbid condition with comorbid condition (renal insufficiency; pregnancy) 4. Number and percentage of uncomplicated patients prescribed drugs without trying a lifestyle change 5. Number and percentage of patients prescribed preventive treatment	Patient’s and provider’s experiences, as well as practice deviations and reasons for: prescribing, as per guide trialling lifestyle changes before drugs prescribing preventive drugs
Lifestyle modification	6. Recording of smoking status and staff response	Patient’s and provider’s experiences, as well as practice deviations and reasons for: counselling patient (with pictorial tool) for lifestyle change and smoking cessation estimating and use of 'target weight' for patient counselling
Follow-up and adherence	7. Number and percentage of patients adhering to follow-up visits (in first 9 months) 8. Number and percentage of patients examined (clinical/ laboratory) on follow-up visits 9. Number and percentage of patients referred for expert check-up and/or complication and/or severe drug reaction	Patient’s and provider’s experiences, as well as practice deviations and reasons for: patient adherence to follow-up visits (include retrieval) staff adherence to care during follow-up visit referrals (for example, side effects)
Material inputs		Patient’s and provider’s experiences, as well as practice deviations and reasons for: maintaining uninterrupted inputs coping with input gaps and/or challenges

**Box 2.** Logic model for the intervention

Intervention inputs	Intervention process and actions	Intended		
		Practice change	Outputs	Health outcome
Case management desk guide and lifestyle counselling tool Training of doctors and allied staff (on full care package) Supplement drugs, equipment and supplies (digital blood pressure apparatus, glucometer, and strips)^a^ Recording forms^a^	Screen/diagnose^a^ Prescribe anti-diabetic drugs Identify co-morbid condition and treat Counsel for lifestyle modification Follow-up care, including retrieval	Providers practise programme protocols to: Screen, diagnose, treat, counsel, follow-up, and report as per programme protocol Patients practise: Follow-up visits Treatment Lifestyle changes (as counselled)	Patients gets: Screened and diagnosed as per programme protocol Prescribed right drug/ dose Counselled for lifestyle change Followed-up and treated for continued care	Reduction (0.5%) in mean HbA1c Increased proportion achieving HbA1c <7%. Reduction in complications related to diabetes

^a^Inputs/ practices kept same in intervention and control arms.

Care providers used chronic disease cards to record clinical care data for the trial patients at 14 public health facilities. The quality of data were ensured, by programme and research staff providing additional training to providers on record-keeping, conducting careful monitoring of data completeness, and by querying any data that appeared implausible. The patient disease card data, collected from October 2014–﻿August 2016, were entered into SPSS (version 17.0). Data were single entered and the quality was assured by data-entry checking at regular intervals to minimise error rates.^6^ The frequency distribution of the data were analysed and cross-tabulated by sex, and by intervention and control.

A preliminary analysis of the quantitative data, collected up to that point, was performed in January 2016. This preliminary analysis was used to identify protocol deviation, and inform the design of semi-structured interviews with care providers and patients at selected intervention sites.

### Facility and participant selection

Four facilities that deliver primary health care — two rural health centres and two urban sub-district hospitals — were randomly selected to understand intervention delivery and experience in rural and urban settings. At each facility, one doctor and one clinic assistant were interviewed.

**Figure 1:**
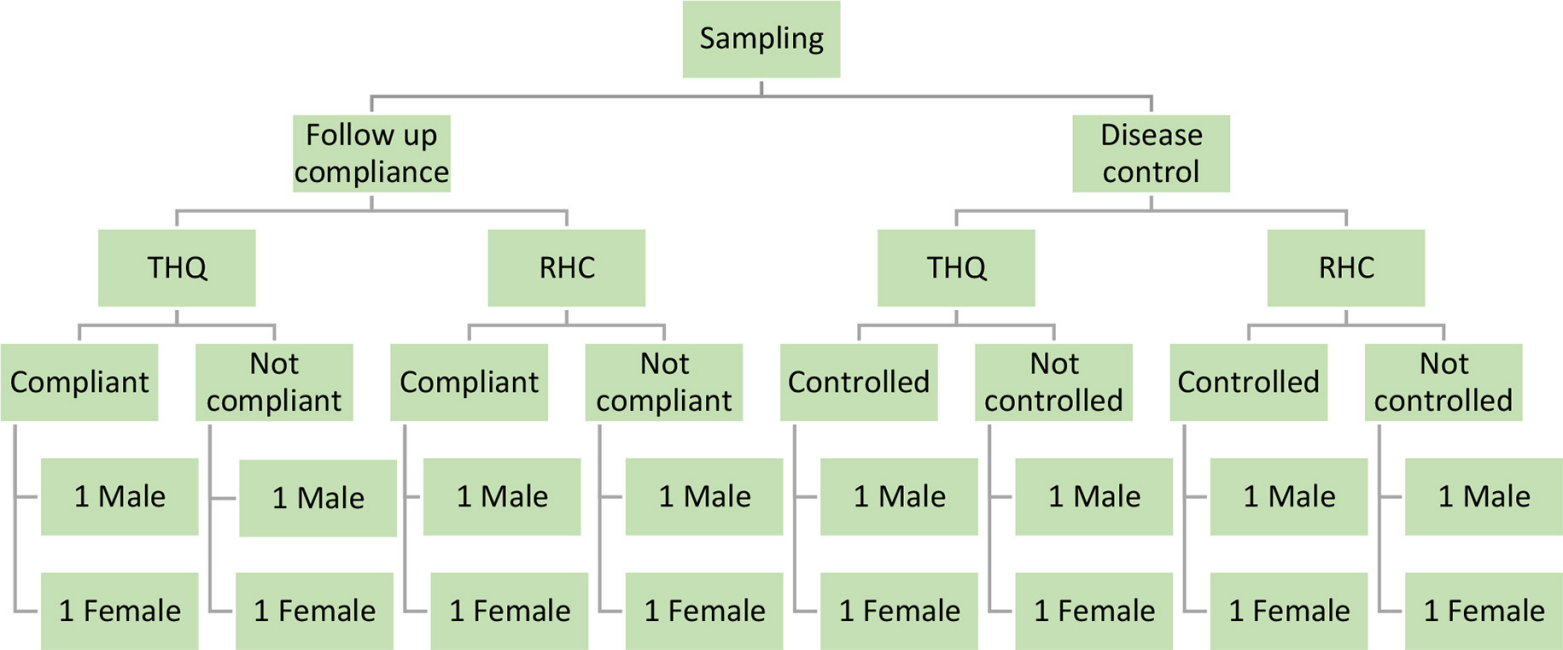
Sampling for interviews. RHC = rural health centre. THQ = Tehsil headquarters.

The facilities were organised into two pairs; one rural health centre and one sub-district hospital per pair. The quantitative data were used to purposively sample patients. In one pair, eight patients (four per facility) were selected according to their adherence to follow-up visits (≥6 follow-up visits in 9 months). Of the four patients in each facility, two (one male and one female) had adhered to follow-up visits and the other two (one male and one female) had not. In the second pair of facilities, another eight patients (four per facility) were selected according to the extent of diabetes control (fasting blood glucose [FBG] ≤125 or random blood glucose [RBG] ≤200). Of the four patients in each facility, two (one male and one female) had controlled their blood sugar levels and the other two (one male and one female) had not (see Figure 1).

All patients who met the criteria described above were identified from the routine data. Patients from the list of those who met the criteria were randomly selected and the clinic assistant was requested to contact them to arrange an interview. With health facility staff facilitation, all potential participants agreed to be interviewed.

Semi-structured interviews were conducted with providers and patients from April–July 2016. The interviews were conducted using Skype call technology and lasted for around 30 minutes. The interviews were conducted in a segregated space to ensure confidentiality and the interviewee was assured that the data would be kept confidential and anonymous. All interviews were recorded. A research assistant was also present at the clinic, to assist the patient with the required technology. An Islamabad-based female member of the research team with experience of qualitative interviewing conducted all the interviews in Urdu. Providers and patients were located within the health facilities in locations that provided confidentiality. The technology was set up by members of the field research team, while the interviewer was located in Islamabad. This format was adopted owing to time and budget constraints for the care providers and the research team, issues concerning the safety of researchers and participants during the data collection period, and gender-related access and adherence issues. Participants provided consent to the interviews verbally and this was recorded.

The transcripts were anonymised and identification keys kept separate from the data. The researcher who collected the data transcribed it in Urdu. Then another researcher checked the transcripts, while listening to the recording. A framework approach was used to analyse the data.^18^ Two members of the team familiarised themselves with the data by reading the transcripts, and identified initial codes. They developed a thematic framework, which was based on five a priori intervention components and emergent issues, and applied it to the data^19^. At this point, data that were included from the transcripts were translated into English by the researcher who collected and transcribed the data, and the quality of the translations were checked by other members of the research team (further information available from the authors on request).

The trustworthiness of the data was ensured by triangulating it from different sources; for example, providers and clients. During the writing process, key findings and quotations were continuously checked against the original transcripts to ensure consistency. Ongoing discussion and agreement by the research team framed the analysis process.

The quantitative and qualitative data were analysed and the findings were integrated. In some cases, the research team had further queries and re-contacted responders in the qualitative study by telephone.

## Results

The major findings are presented below, under five key task categories.

### Screening and diagnosis of diabetes

Between 0.05% and 0.1% of outpatients were diagnosed as having diabetes across the intervention and control piloting sites. This was lower than might be expected.^20^ The data is not available to estimate the proportion of outpatients who were screened, tested and found to have high blood glucose levels.

About 60% of the diagnosed patients were overweight. This suggests that, as proposed, patients were screened on the account of appearance (of being overweight) and symptoms of diabetes. One of the responding doctors summarised his experience as follows:

*'Patients do report with different set of symptoms and signs, therefore we rely on a range of indications for diabetes.'* (Doctor RHC)

At the time of diagnosis 417 (84%) patients were administered both RBG and FBG; the remaining (16%) patients were administered only one of the two tests (RBG 5%; FBG 11%). A total of 2–3 consecutive facility visits were required for a patient to get examined and diagnosed. The facility staff and patients did not report any financial or social constraint to coping with this requirement:

*'The doctor got a few tests done for diagnosing my diabetes, for which I visited the facility three times. I did not pay any fee for these tests.'* (Female patient THQ)

The definition and understanding of FBG testing was found to vary among patients; for example, some patients reported taking a cup of tea (but no breakfast) before coming for the FBG testing.

Urine protein testing was used to screen patients for renal impairment. Urine protein was found to have been tested for all 495 diabetes patients. A total of 192 (39%) of the patients were found to have some degree of proteinuria: 134 (27%) had mild proteinuria; 58 (12%) had moderate or severe proteinuria, which is associated renal compromise.

The facility staff indicated that the use of dipstick made the urine-protein screening more feasible and quicker for staff and patients. Neither staff nor patients reported any financial, social or logistic constraints in getting the urine tested. For example:

*'Urine dipstick is more manageable for our situation, i.e. heavy patient load.'* (Allied staff THQ)

The core demographic measures (age, sex) and clinical parameters (for example, weight and blood pressure) were found recorded for all registered patients. The data show that about 60% of the registered diabetes patients were women. The facility staff and patients reported no social and/or logistic constraint in: (a) getting a patient clinically examined and recorded; and (b) patients’ sharing their phone numbers, when the purpose was explained to them. However, most female patients shared the phone numbers of their male family members (husband, son, and so on) for the facility records:

*'Female patients usually prefer sharing the phone number of a male family member; as this is socially more acceptable* [in this cultural setting]*.'* (Allied staff RHC)

When asked about the possible reason for more female patients, the facility staff mentioned minimal opportunity cost (that is, lost earning) as a main reason for more female patients, rather than more women suffering from the disease.

### Prescriptions

All patients were found to have been prescribed medication at the time of diagnosis; no patient was given lifestyle modification trial before prescribing the drugs. Prescribing drugs at diagnosis seems to stem from patient expectation and the provider’s routine, which is to prescribe everyone at the time of diagnosis:

*'It is difficult to inform a patient about diagnosis and not prescribe any medicine; the patient is likely to switch to another source of medical care.'* (Doctor RHC)

Table 1 shows that the intervention-site patients were more often prescribed the correct medicine, as per programme protocols (namely, that metformin should be prescribed as first-line drug, unless associated with protein in urine), compared with the control sites: 49% and 131% more often among patients without and with proteinuria respectively.

**Table 1.** Prescriptions for diabetes

Arm	Treatment without associated proteinuria						Treatment with associated proteinuria					
	Biguanide		Others		Total		Sulphonylurea		Others		Total	
	*n*	%	*n*	%	*n*	%	*n*	%	*n*	%	*n*	%
Intervention	141	63.2	82	36.8	223	100	14	53.8	12	46.2	26	100
Control	91	42.5	123	57.5	214	100	7	23.3	23	76.7	30	100
Total	232	53.1	205	46.9	437	100	21	37.5	35	62.5	56	100

Variation in prescription practice seems to be a blend of medical and non-medical considerations. Non-medical reasons for drug preferences were provider preference, patient profile, and drug cost and availability. Some of the prescription variations reflect more the provider’s perceived balance between the medical and non-medical considerations:

'*Some patients do prefer taking expensive medication (by multinational pharmaceutical companies) or medicine being used by other family member or friend.'* (Doctor RHC)

At time of registration, around 10–12% of patients with diabetes were labelled hypertensive and prescribed antihypertensive drugs, whereas another 12–13% were found with raised BP but not labelled and not treated for hypertension. Interviews with staff indicated inconsistencies in labelling a patient with coexisting diabetes and hypertension, and also in recording antihypertension drugs, especially if not provided from the facility drug stock:

*'Patient records are sometimes used as evidence of drugs dispensed by the facility; so we tend to record only the drugs provided from the facility stock.'* (Doctor RHC)

Table 2 shows that the majority of eligible patients were not prescribed any preventive drug (a statin and/or aspirin unless a contraindication), even when available in the facility stock. Interviews with doctors at intervention facilities indicated the challenge of selecting eligible patients for prescribing preventive treatment. Some also suggested considering universal prescription of preventive drugs, with as few exclusions as possible:

**Table 2.** Preventive medication

Arm	Eligible			Treated	
	Raised blood pressure	Smokers	Total	*n*	%
Intervention	60	37	97	5	5.1
Control	68	28	96	13	13.5
Total	128	65	193	18	9.3

*'Heavy outpatient load makes it difficult to apply any complex care protocol such as eligibility for preventive medication.'* (Doctor THQ)

### Lifestyle modification

The average reported duration of lifestyle counselling session at the time of registration was about 10 minutes, whereas subsequent counselling sessions were shorter, at around 5 minutes. Women were reported to show more interest in lifestyle counselling, including weight reduction.

At the time of registration, around a quarter of male patients and 5% of female patients were reported smokers. At the time of outcome measurement, 9 months after registration, only one smoker reported having quit smoking. Staff and patients indicated that staff prioritised counselling on diet and exercise, with less attention to smoking cessation.

Comparing weight at registration and the sixth follow-up visit, the intervention was found to have maintained or reduced the body weight of 69% of patients, while 31% of patients showed an increase in body weight. The change in weight indicates some possible lifestyle changes involving diet and/or exercise. The concept of target weight was also reported to be well perceived by staff and patients, as it set a clear target for weight change.

Patients reported challenges in managing their daily walk (30 minutes) owing to lack of access to a walking place and difficulty in finding time from other responsibilities. Women also reported an additional cultural challenge to their walking practices of needing a male member to accompany them. Some women managed by walking within their household boundaries, such as in courtyards and roof-tops.

Data suggest that the change of diet was a complex social phenomenon, rather than just a matter of health need or priority. The ability to change the family dietary practices seems to be associated with who in the household has suggested the dietary change. Male wage-earners were more likely to get the family diet changed compared with a female homemaker or an older person. A few women also reported managing their salt and/or oil reduction by taking their portion out, then adding salt or oil to the food for the rest of the family members. One commented:

*'My mother-in-law decides the menu and recipe for family meals; any dietary change without her consent is not easy.'* (Female patient THQ)

### Follow-up and adherence

**Figure 2:**
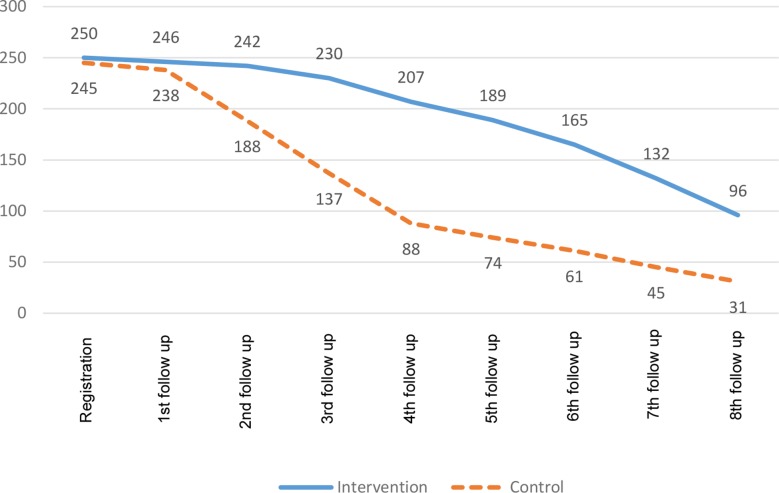
Patient attrition in intervention and control arms

Patients at intervention sites adhered more to the first eight monthly follow-up visits (75%; *n* = 1507/2000) as compared with the control sites (44%; *n* = 862/1960). This difference seems more marked during the first five monthly follow-up visits, and becomes less pronounced in subsequent months (see Figure 2).

Interviews with staff and patients indicated free drugs and tests to be the incentives for patients’ continued facility visits:

*'I come to the health centre to get free examination and drugs.' (*Patient RHC)

The intervention sites implemented a three-tray system, whereby patient treatment cards were moved to the next tray when they attended, while those not attending remained and were made a phone recall contact. The better adherence to follow-up visits at intervention sites indicates the value of this patient adherence monitoring and retrieval system. The allied staff reported no challenge in their reminding male and female patients about their follow-up visit. However, staff suggested using standardised messages for typical scenarios, such as reminders to patients with delayed follow-up visit, and reminders for lifestyle changes:

*'Useful application of mobile technology for patient adherence may be sustained and possibly expanded for other disease conditions.'* (Paramedic THQ)

Many individuals faced the challenge of being consistent in attending, taking life-long medication and lifestyle change. More than half of the interviewed staff and patients mentioned patients using supplementary care from traditional and/or faith-based sources in the hope of finding a cure, rather than accepting the explanation of their diagnosis and the prospect of lifelong treatment:

*'On my friend’s suggestion, I did try water from a holy spring in hope of getting cured.'* (Patient THQ)

The review of more than 2350 patient follow-up visit records, showed universal staff adherence to the clinical assessment (for example, BP, weight) and blood glucose testing requirements. However, the staff did indicate the importance of uninterrupted supplies (for example, glucose testing strips) and equipment maintenance (for example, BP apparatus, weighing scale, and so on) for them to be able to examine patients properly. No patient reported any significant financial or social challenge in attending their follow-up appointments.

During the first 9 months of treatment, 41 patients (8%) visited a secondary level hospital; 19 (46%) of these visits were for cardiovascular disease-related conditions; and 34 (83%) were admitted for 1 or more nights. Seventeen (41%) of the 41 reported visits were made to a public hospital, and 24 (59%) were made to a private hospital. When a patient has a severe medical condition, the doctor and the patient’s family judge which hospital and when to attend, taking into account financial and other considerations. Even referral for a routine specialist examination (for example, an eye examination) at a hospital can be a challenge, which may result in lower patient compliance:

*'It took three hospital visits for me to get checked by eye specialist, because I went without prior information and appointment.'* (Patient THQ)

### Material inputs

Project records and staff interviews show that the drugs, laboratory supplies, and equipment were effectively channelled through the respective provincial programme and district offices. The facility staff and patients did not report any complaints regarding distribution, quality, or use of programme provided drugs and supplies. However, the facility staff mentioned the challenges of maintaining equipment and non-inclusion of the cholesterol-lowering drug (statins) in the list of drugs for the public facilities to purchase.

## Discussion

### Summary

Overall, the intervention components were found to be feasible to implement for the providers and were acceptable to patients. The intervention led to improved prescribing practice and better attendance for follow-up visits. The data on lifestyle counselling and change were inconclusive and need further emphasis in future. Two important health system factors that helped to make the diabetes care available and affordable for patients were use of the chronic disease card, and regular supply of free drugs and tests.

The intervention showed improved patient glucose control outcomes, although this was not statistically significant after adjusting for cluster effects and potential confounding variables. A limiting factor on the size of effect may be the reduction of follow-up from 18 to 9 months, which was owing to operational and time-constraints. The results need to be assessed again with a longer treatment follow-up and refinements made following a process evaluation of the intervention.

### Strengths and limitations

A pragmatic approach to data collection was adopted, which had both strengths and weaknesses. The strengths were that, by utilising routine data, data collection costs were minimised and the quantity of data available for analysis was maximised. However, there were some variables that were not available such as education level and occupation. There is a lack of literature evaluating the use of Skype interview in applied health research, such as participant discomfort with technology, or technical failure. However, the authors' experiences were broadly positive and utilisation of Skype technology allowed them to conduct the research according to the planned protocol and with an expert interviewer.

### Comparison with existing literature

The international guidelines^21^ recommend FBG as an essential test for diagnosis and follow-up of diabetes. The present study indicates that testing FBG is feasible at the time of diagnosis. However, FBG testing during the follow-up seems to add to the patient and staff efforts without necessarily adding to the quality of clinical care. Global literature suggests that RBG can be used as a reasonable test to check the glycaemic control of undertreatment of patients in resource poor settings.^22–24^ Therefore, the authors recommend the use of RBG (instead of FBG) to simplify the follow-up requirements for patients and staff, without compromising the quality of care.

This study showed that using a dipstick for testing proteinuria is feasible for primary healthcare settings. This seems in-line with experiences elsewhere; for example, Patel *et al* ^25^ stated that urine dipstick is feasible and reduces staff workload in routine primary care. Given that 40% of patients with diabetes had some degree of proteinuria, and the degree of proteinuria does affect the prescription, the authors recommend universal urine dipstick testing of patients with diabetes at primary healthcare facilities.

This study found more diagnoses of diabetes among women than men. One explanation could be higher prevalence of diabetes in women than men. The literature suggests occupation, education, socioeconomic status, marital status, obesity, and increased waist circumference to be the major reasons for possible high prevalence among females.^26–29^ However, another plausible explanation is higher service utilisation among women.^30–31^ In this case, the data were inconclusive as to why more women than men were diagnosed.

Prescription variation among intervention doctors indicates that, in addition to guideline and training, staff enhanced supervision is needed to achieve universal adherence to the prescription protocols. The literature shows evidence of doctors: (a) advocating specific brands of medicine in response to promotion by pharmaceutical companies;^32–34^ and (b) prescribing drugs in response to patient preferences, such as drug cost and perceived quality.^35–36^ In the present study, the availability of drugs influenced the staff and patient response, and the authors recommend programme measures to ensure supply of essential drugs at public health facilities.

The very low prescription of preventive medicine, even when made available, indicates the need to simplify the prescription protocols. The recent literature also proposes universal prescription of preventive medicine, with some exclusion criteria.^37^ The authors recommend adapting the protocols for prescribing preventive medication, in line with criteria-based exclusion approach.

The authors found helpful, as reported elsewhere,^38–40^ the use of mobile phone to remind patients about their follow-up appointments. Nevertheless, 25% of patients still did not adhere to their follow-up appointments. The King’s Fund^11^ reported similar challenges of chronic disease patients’ non-adherence to the follow-up visits. Others report higher adherence to the follow-up visits when patients were offered transfers to another health facility of their choice or convenience^41–42^ and prescription refills.^43^ The authors recommend that the NCD programme contextualise and evaluate such innovations for potential future scaling.

Lifestyle change is a complex sociocultural phenomenon that is mediated by family, community, and gender dynamics.^44^ This study found that women faced greater challenges than men in making changes to their diets and physical activity levels.^39,40,45,46^ This could relate to a number of sociocultural factors; for example, decision-making power, gendered division of labour, and traditional home-based roles.^47,48^ However, most literature relates to high-income settings, and interventions often rely on behaviour change techniques^43–45^ that have emerged out of Euro-American psychological theory. The authors recommend research to understand how to modify the lifestyle of patients with diabetes in developing country settings, with interventions focusing on both individuals and communities.

### Implications for research and practice

By utilising mixed methods for a process evaluation, the authors were able to develop an in-depth understanding of the perceptions of providers and patients of what was delivered, to whom, and why. Some challenges were identified relating to continued diabetes care and lifestyle modification, and the authors have proposed recommendations for better diabetes care in Pakistan.

This study shows that integrating diabetes management into routine primary care is feasible and acceptable. Integrated diabetes and related disease care can lead to improved assessment, diagnosis, prescription practices, and adherence to follow-up appointments in a low-income country setting, with promising results. The authors recommend service delivery refinement and continued research for achieving optimal diabetes care.
